# Social Robots and Sensors for Enhanced Aging at Home: Mixed Methods Study With a Focus on Mobility and Socioeconomic Factors

**DOI:** 10.2196/63092

**Published:** 2024-11-25

**Authors:** Roberto Vagnetti, Nicola Camp, Matthew Story, Khaoula Ait-Belaid, Suvobrata Mitra, Sally Fowler Davis, Helen Meese, Massimiliano Zecca, Alessandro Di Nuovo, Daniele Magistro

**Affiliations:** 1 Department of Sport Science School of Science and Technology Nottingham Trent University Nottingham United Kingdom; 2 Department of Computing & Advanced Wellbeing Research Centre Sheffield Hallam University Sheffield United Kingdom; 3 Wolfson School of Mechanical, Electrical, and Manufacturing Engineering Loughborough University Loughborough United Kingdom; 4 Department of Psychology Nottingham Trent University Nottingham United Kingdom; 5 Faculty of Allied Health and Social Care Anglia Ruskin University Chelmsford United Kingdom; 6 The Care Machine Ltd Potterhanworth United Kingdom

**Keywords:** older adults, motor difficulties, socioeconomic status, social assistive robots, monitoring technologies, mixed methods

## Abstract

**Background:**

Population aging affects society, with a profound impact on daily activities for those of a low socioeconomic status and with motor impairments. Social assistive robots (SARs) and monitoring technologies can improve older adults’ well-being by assisting with and monitoring home activities.

**Objective:**

This study explored the opinions and needs of older adults, including those with motor difficulties and of a low socioeconomic status, regarding SARs and monitoring technologies at home to promote daily activities and reduce sedentary behaviors.

**Methods:**

A mixed methods approach was used, with 31 older adults divided into 3 groups: those of a low socioeconomic status, those with motor difficulties, and healthy individuals. Focus groups were conducted, and they were analyzed using thematic analysis. Perceived mental and physical well-being were assessed using the 12-Item Short Form Health Survey, and attitudes toward robots were evaluated using the Multidimensional Robot Attitude Scale.

**Results:**

The results identified 14 themes in four key areas: (1) technology use for supporting daily activities and reducing sedentary behaviors, (2) perceived barriers, (3) suggestions and preferences, and (4) actual home technology use. Lower perceived physical well-being was associated with higher levels of familiarity, interest, perceived utility, and control related to SARs. Lower perceived psychological well-being was linked to a more negative attitude, increased concerns about environmental fit, and a preference for less variety. Notably, older adults from the low–socioeconomic status group perceived less control over SARs, whereas older adults with motor difficulties expressed higher perceived utility compared to other groups, as well as higher familiarity and interest compared to the low–socioeconomic status group.

**Conclusions:**

Participants indicated that SARs and monitoring technologies could help reduce sedentary behaviors by assisting in the management of daily activities. The results are discussed in the context of these outcomes and the implementation of SARs and monitoring technologies at home. This study highlights the importance of considering the functional and socioeconomic characteristics of older adults as future users of SARs and monitoring technologies to promote widespread adoption and improve well-being within this population.

## Introduction

### Background

Population aging is a significant phenomenon with far-reaching implications for both the economy and society. Worldwide, older adults constitute approximately 13% of the population, with this proportion increasing by 3% annually [1], whereas projections for the United Kingdom suggest that, by 2050, a quarter of the population will be aged ≥60 years [2]. This demographic shift introduces new challenges, notably, a heightened need for health and social services [2] as aging is associated with a reduction in physical and cognitive functioning and an increase in frailty conditions [3-5]. Frailty conditions lead to a reduction in an older person’s ability to carry out activities required to live independently, known as activities of daily living (ADLs) [6,7]. To counteract this, there is a global increasing interest in age-friendly environments that may include social or technological innovations [8] that support older adults in remaining independent in their daily lives. In this paper, “daily activities” refer to ADLs. These activities can be physical, such as those needed to manage physical needs, or complex, referring to tasks necessary for independent living within the community [8].

The ability to perform ADLs tends to diminish gradually starting from late middle age and during seniority [9,10] due to a reduction in fundamental physical abilities [11-13]. One method of promoting healthy aging and independence in older age is maintaining an active lifestyle [14]; however, older adults often find themselves spending most of their time engaged in sedentary behaviors [15]. Increased sedentary behavior is associated with a higher risk of mortality and adverse health outcomes [16]. In contrast, the performance of daily physical activities is reported to be linked with various indicators of well-being, including improved mental health [17], reduced risk of chronic diseases [18], a reduction in long-term assistance needs [19], reduced hospitalization rates [20], reduced carer burden [21], and reduced mortality [22]. Thus, national and international recommendations suggest decreasing older adults’ sedentary time and increasing time spent in physical activities [23].

In addition to declining physical abilities, factors such as lifestyle, health indicators, social isolation, and socioeconomic status—including household income—can affect the ability of older adults to perform daily activities [24]. Within the socioeconomic context, higher socioeconomic status is associated with better health status [25] and decreased frailty conditions [26], and notably, a lower socioeconomic status is linked to a greater need for personal, instrumental, and environmental support [27]. Therefore, individuals of a low socioeconomic status may have specific needs that ought to be considered when designing interventions to promote healthy aging.

There is a current trend of interventions aimed at improving quality of life for older adults consisting of adapting their environment to enable them to live as independently as possible for as long as possible, known as “aging in place” [28]. Aging in place refers to older adults’ capacity to remain in their own homes and communities securely, independently, and comfortably irrespective of their age, financial resources, or functional limitations [29]. Through these interventions, support is provided to enhance the well-being and independence of older individuals while reducing health care costs [30,31]. Importantly, because of the high levels of variation among the older population, care provision needs to encompass a wide range of options, with home-based technology serving as a potential tool to reduce the daily burden on primary carers [32]. Within this framework, smart home technologies, such as monitoring systems and social robots, offer promising solutions for helping older adults maintain independence and age in place, particularly in the context of an aging population and a shortage of care workers [33]. The literature indicates that the integration of home modification strategies into smart homes to monitor daily activities and health is viewed as a key factor in enabling successful aging in place [34]. Systems incorporating these technologies use artificial intelligence models to understand older adult users and make informed decisions, with a primary focus on activity assistance and recognition [35].

Social assistive robots (SARs) have proven to be a valid method of supporting older adults and people with clinical conditions [36,37]. The literature indicates a strong interest in understanding the psychological dimensions of human-robot interaction [38]. SARs offer significant potential to improve the quality of life of older adults by providing physical assistance with ADLs [6,39] and carrying out cognitive assessments [40], which are fundamental aspects of supporting “aging in place” solutions [41,42]. The use of these technologies to promote the well-being and healthy behaviors of older adults has increased in recent years. However, it has been noted that their design may not adequately meet the diverse needs and capabilities of all users due to the current limitations in involving individuals with varying characteristics and of various socioeconomic backgrounds in a user-centered design framework [43]. Older adults often embrace robots, sometimes more than their younger counterparts [44]. However, these relationships between older adults and robots are often complex, with the acceptability of robots differing based on financial availability [45], previous experience [46], and perceived usefulness [6]. Discrepancies between the needs of users and the solutions currently provided by SARs have the potential to diminish their adoption [47]; however, acceptability could be improved by tailoring them to users’ needs and issues [48].

Alongside SARs, other technologies can be used to detect specific activities taking place within a designated room [49], allowing for the adoption of more specialized care and home adaptation strategies [50]. One strength associated with these technologies is their capacity to provide objective measurements within the domestic environment [51], thus informing decision-making among statutory providers, family carers, and older people. Various types of sensors, both environmental and wearable, have been widely used in the literature to evaluate and recognize older adults’ daily activities [52]. Smart home systems have been widely used to assess daily activities. The most common methods involve environmental technologies, with infrared motion sensors and contact sensors being the most frequently used [53]. Data collected from sensors can be used to detect routines, identify deviations from typical daily activities [54], and monitor potential health issues [34]. Given their potential in the assessment of daily activities and various health-related issues, monitoring technologies installed in older adults’ homes play a crucial role in the aging-in-place framework [34] and prompt a discussion on the significance of incorporating users’ opinions to enhance the design and usability of these sensors [52].

### Objectives

This study aimed to understand the perspectives of older adults regarding the use of SARs and monitoring technologies in their domestic environment to reduce sedentary behavior and promote daily physical activities. In addition, through the inclusion of older adults with motor difficulties and those of a low socioeconomic status, this study aimed to recognize the considerable role that these aspects play in influencing individuals’ daily activities, needs, and well-being.

## Methods

### Study Design

This study used a mixed methods approach, with qualitative data gathered during focus groups and quantitative measures collected through the questionnaires described in the Measurements section. This study used a parallel design in which the collection of both quantitative and qualitative data occurred simultaneously but the data were analyzed separately [55]. This design aimed to create distinct sets of data that informed each other and were later integrated to provide a more comprehensive understanding of the overall topic. The questionnaires were web-based, and reporting of these methods and results was in line with the Checklist for Reporting Results of Internet E-Surveys [56] (Multimedia Appendix 1).

### Procedure

This study included older adults from the United Kingdom recruited from June 2023 to September 2023. Participants were contacted through social media, email lists and advertisements from charity groups, care homes, and universities. To be eligible for this study, all participants had to be aged ≥65 years. Participants were divided into three groups according to the following criteria: (1) older adults with a relatively low income, as defined by the most recent UK statistics [57], were considered as the *low–socioeconomic status group*; (2) older adults with reported motor difficulties and needing physical support to move were considered as the *motor difficulties group*; and (3) older adults not in relative poverty, without physical difficulties, and able to live independently were considered as the *healthy group*.

Participants were asked to complete an online survey at their own pace and then invited to take part in one of the focus groups. The survey collected demographic information, health information, and responses to the questionnaires described in the Measurements section. Focus groups were planned as qualitative data collection that included participants’ reported experiences, with ongoing analysis conducted until saturation was achieved [58]. Focus groups were used as a qualitative research technique due to their advantages in fostering interaction to generate ideas and gain deeper insights into participants’ beliefs, attitudes, motivations, and perceptions [59]. This approach allows for a shared understanding of daily life and encourages the use of everyday language. The diverse responses provide a richer understanding of the topic, enabling connections to be made and participants’ viewpoints to be continually re-evaluated [59]. These methods were used to obtain both qualitative and quantitative data from participants. A total of 40 participants joined the study. In total, 22% (9/40) of the participants withdrew for various reasons, resulting in 31 participants who remained involved.

### Ethical Considerations

Before taking part in the study, participants were provided with information regarding the study and asked to sign an informed consent form. This study was approved by Nottingham Trent University Institutional Human Research Ethics Committee (ID 1726544) and was conducted according to the principles established by the Declaration of Helsinki. The participants were reimbursed for their travel expenses. Data were anonymized.

### Focus Group Structure

**Figure 1:**
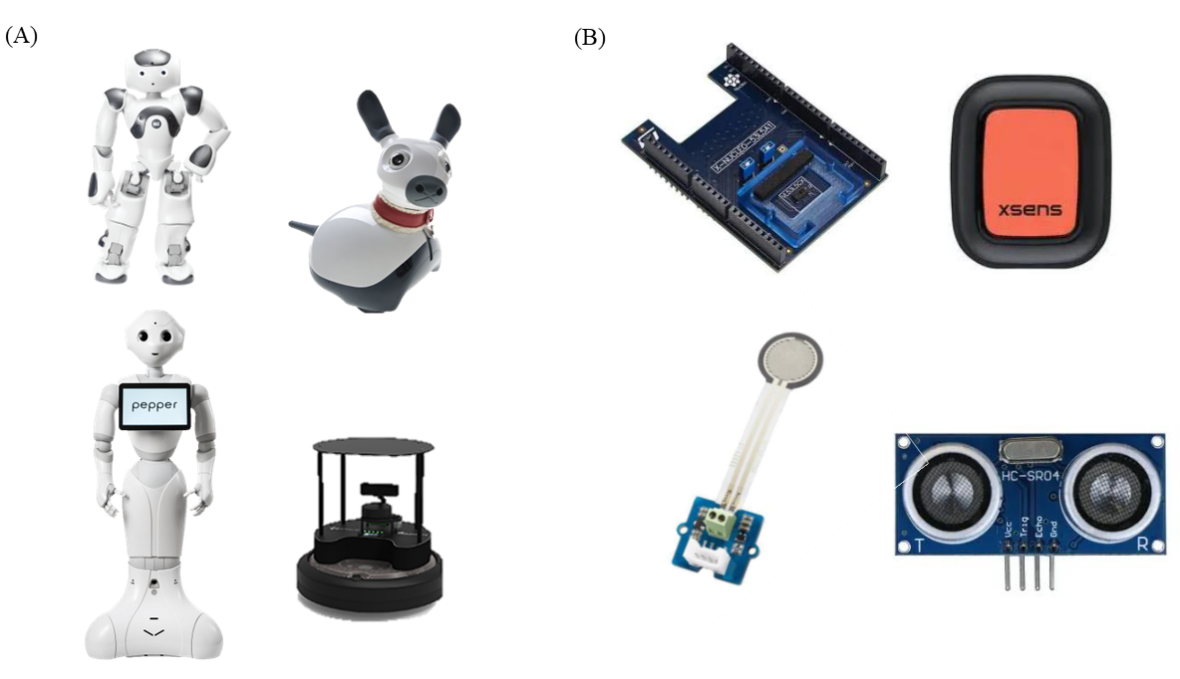
(A) Social assistive robots. From left to right: NAO, MiRo-E, Pepper, and TurtleBot. (B) Monitoring technologies presented to the participants before the discussion. From left to right: light detection and ranging, Xsens, pressure sensor, and ultrasonic sensors.

Participants engaged in focus groups comprising 6 to 8 individuals to explore how SARs and monitoring technologies could support older adults in reducing sedentary behavior and enhancing their daily activities. Before the discussion and instructional phase, participants were informed about the focus groups’ objectives and discussion rules. A presentation was organized to introduce the 3 main topics: ADLs, SARs, and monitoring technologies. Daily activities could range from the basics to more complex activities, leading to a distinction between basic and instrumental activities [60,61]. For instance, basic daily activities encompass bathing, feeding, or mobility, whereas instrumental activities comprise tasks such as housekeeping or managing medications and finances [62,63]. Basic and instrumental ADLs were presented through a Microsoft PowerPoint presentation, followed by a live demonstration of various SARs, as depicted in Figure 1A. The showcased SARs—NAO, Pepper, MiRo-E, and TurtleBot 4—represented a diverse range of types, functions, dimensions, movements, and characteristics. The rationale for selecting these robots was to provide participants with examples of widely used SARs, showcasing the widest possible range of variations and features. This selection aimed to enable participants to provide concrete ideas and associations related to these technologies during the focus groups. NAO and Pepper were chosen as humanoid robots, both equipped with a variety of sensors and hands capable of gripping objects. However, they differ in dimensions (58 and 120 cm, respectively), types of interaction, and movement style. A key difference is the ability of Pepper to interact using a tablet on its chest, which is not a feature of NAO. Similarly, Pepper has built-in omnidirectional wheels to maneuver itself, whereas NAO is able to walk in a more human fashion due to having legs and feet containing specific motors and joints. MiRo-E was selected to showcase a more minimalistic appearance with an animal-like design. TurtleBot 4 was chosen as an objectlike robot with a design similar to those commonly used at home. It features a differential drive base and sensors for perception, offering versatility. The research team described each SAR’s main features and functions, allowing participants to interact with the SARs during the session. Monitoring technologies were explained through a PowerPoint presentation, accompanied by a physical display of various sensor types to participants (Figure 1B), including ultrasonic, light detection and ranging, pressure sensor, and Xsens. The presentation aimed to equip participants with sufficient information to offer coherent suggestions on the topic and provide a more tangible understanding of the current capabilities of SARs and monitoring technologies. The presentations typically lasted between 20 and 30 minutes in total. This segment was followed by the focus group discussion, which took the form of a semistructured interview. Participants were asked about challenges they face in their daily activities, how SARs and monitoring technologies could assist them in reducing sedentary behavior and in daily activities, and their suggestions and concerns regarding the use of SARs and monitoring technologies in the home. The discussions were audio recorded and then transcribed verbatim. The focus groups typically lasted approximately 90 minutes (range 83-101 minutes) with a 15-minute break.

### Measurements

The 12-Item Short Form Health Survey [64], a short form of the Medical Outcomes Study 36-Item Short Form Health Survey [65], is a 12-items questionnaire that assesses people’s perception of their general health according to physical and mental health components. A norm-based standardized score is calculated [66] leading to a physical component score and a mental component score.

The Multidimensional Robot Attitude Scale (MdRAS [67]) is a questionnaire developed to assess people’s attitudes toward domestic robots investigating 12 dimensions: familiarity, interest, negative attitude, self-efficacy, appearance, utility, cost, variety, control, social support, operation, and environmental fit. The questionnaire is composed of 49 items on a 7-point Likert scale and reflect people's expectations regarding future interactions with real SARs.. Participants were provided with images of SARs before completing the questionnaire.

### Thematic Analysis of Focus Groups

The focus groups underwent a comprehensive thematic analysis following an inductive approach [68] using NVivo (QSR International). This method encompasses several key phases, beginning with familiarization with the data followed by the generation of codes, linking these codes to overarching themes, and subsequently reviewing and defining these identified themes. The final step involves compiling a comprehensive report summarizing the outcomes of the analysis. The fit between the codes and statements was reviewed in a series of research team meetings after each focus group during which codes were revised leading to possible changes, splitting, or conceptual expansions [69,70]. The reliability of the codes was then assessed through cross-coding comparison with a small portion of the overall transcripts. To this end, 2 independent research team members who had not previously been involved in the coding processes were introduced to the coding manual and asked to independently code a sample of 10% of the transcripts, indicating strong agreement between the two coders (*k*=0.81). Codes were then grouped and refined into themes during a series of meetings, and a final report was compiled. Saturation was considered achieved based on a code frequency count approach, with a stopping criterion established as a new information threshold of ≤5% [58].

### Statistical Analysis of Questionnaires

The statistical analysis should be interpreted with caution due to the small sample size and further evaluated in future studies. A preliminary Shapiro-Wilk test was conducted, indicating that the data did not follow a normal distribution; therefore, nonparametric techniques were used. To assess differences in scores related to physical (physical component score) and psychological (mental component score) well-being measured using the 12-Item Short Form Health Survey, as well as attitudes toward domestic robots assessed using the MdRAS, the groups were compared using the Kruskal-Wallis test. If a significant result was obtained, pairwise comparisons were then further evaluated using the Dwass-Steel-Critchlow-Fligner test. In addition, Spearman correlations were performed to evaluate associations between the physical and psychological dimensions and the various attitudes toward domestic robots.

## Results

After presenting the characteristics of the participants and groups, this section presents the results of the thematic analysis followed by the quantitative statistical results and their joint outcomes.

### Participants

A total of 4 focus groups were conducted with 31 participants divided into 3 groups: low–socioeconomic status individuals, individuals with motor difficulties, and healthy individuals. The groups were balanced regarding age (χ^2^_2_=2.7; *P*=.25) and sex (*P*>.99; Fisher exact test). The groups showed no differences regarding psychological well-being (χ^2^_2_=1.7; *P*=.43); however, significant differences were found on physical well-being (χ^2^_2_=19.7; *P*<.001), where the group with motor difficulties showed lower scores compared to the low–socioeconomic status group (*W*=−5.34; *P*<.001) and the healthy group (*W*=−5.47; *P*<.001). Group statistics are reported in Table 1.

**Table 1:** Description of the participants who took part in the focus groups (N=31).

Variable				Healthy group (n=10)		Motor difficulties group (n=10)		Low–socioeconomic status group (n=11)		Statistical test		
										Chi-square (*df*)		*P* value
**Sociodemographic characteristics**												
	Age (y), median (IQR)		75.5 (70.8-80.3)		71.5 (68.3-75.5)		69.0 (68.0-75.5)		2.7 (2)		.25	
	**Sex, n (%)**									—^a^		>.99^b^
		Male	4 (40)		4 (40)		4 (36)					
		Female	6 (60)		6 (60)		7 (64)					
**Health-related characteristics**												
	PCS-12^c^ score, median (IQR)^d^		52.8 (50.2-53.7)		31.7 (22.2-35.5)		52.7 (51.0-54.0)		19.7 (2)		<.001	
	MCS-12^e^ score, median (IQR)		58.7 (51.4-59.4)		59.1 (39.9-60.7)		55.7 (49.3-58.3)		1.7 (2)		.43	

^a^Not applicable

^b^Fisher exact test.

^c^PCS-12: physical component score of the 12-Item Short Form Health Survey.

^d^The group with motor difficulties had a significantly lower score on the PCS-12 than the other 2 groups.

^e^MCS-12: mental component score of the 12-Item Short Form Health Survey.

### Thematic Analysis of Focus Groups

#### Overview

The thematic analysis resulted in a total of 14 themes divided into 4 key areas: (1) use of technology to support daily activities and reduce sedentary behaviors, (2) perceived barriers, (3) suggestions and preferences, and (4) actual use of technology in the home. Saturation was achieved. For each theme, we report in Table 2 the percentage of related statements made by each group. In the following sections, the type of user who made each comment is indicated in brackets next to the quotes. The full list of comments by participants for each theme is available at the online repository [71]. In addition, for each theme, it is indicated whether it relates to SARs, monitoring technologies, or both.

**Table 2:** Joint table of qualitative and quantitative results. Percentages and absolute frequencies of a theme are reported for each group. Quantitative dimensions with common concepts are reported for each theme.

Theme		Group, n/N (%)			Quantitative attitude
		Healthy individuals	LSE^a^	MD^b^	
**Use of technology to support daily activities and reduce sedentary behaviors**					
	Managing daily activities	13/36 (36)	11/36 (31)	12/36 (33)	—^c^
	Motivating and stimulating older adults	6/26 (23)	8/26 (31)	12/26 (46)	Familiarity^d^
	Providing physical assistance	7/33 (21)	3/33 (9)	23/33 (69)	—
	Continuous monitoring of health, safety, and activities	15/45 (33)	11/45 (25)	19/45 (42)	—
**Perceived barriers**					
	Social support	9/36 (25)	19/36 (53)	8/36 (22)	Familiarity^d^
	Personal factors	17/52 (33)	22/52 (42)	13/52 (25)	Self-efficacy, negative attitude, and social support
	Economic factors	5/18 (28)	9/18 (50)	4/18 (22)	Cost
	Privacy	5/15 (33)	7/15 (47)	3/15 (20)	—
	Spatial issues	2/16 (13)	10/16 (62)	4/16 (25)	Environmental fit
**Suggestions and preferences**					
	Number of devices	6/15 (40)	5/15 (33)	4/15 (27)	—
	SAR physical characteristics	21/57 (37)	14/57 (25)	22/57 (38)	Appearance and variety
	Personalization and remote control	14/39 (36)	10/39 (26)	15/39 (38)	Control^e^ and operation
	Monitoring technology use	7/22 (32)	7/22 (32)	8/22 (36)	—
Actual use		13/38 (34)	14/38 (37)	11/38 (29)	Utility^f^

^a^LSE: low socioeconomic status.

^b^MD: motor difficulties.

^c^No common concepts between the quantitative dimensions and the theme.

^d^The MD group showed significantly higher scores than those in the LSE group (*P*=.005).

^e^The LSE group showed significantly lower scores than those in the healthy group (*P*=.02) and the MD group (*P*=.001).

^f^The MD group showed significantly higher scores than those in the healthy group (*P*=.01) and the LSE group (*P*=.003).

#### Use of Technology to Support Daily Activities and Reduce Sedentary Behaviors

##### Managing Daily Activities (SARs and Monitoring Technologies)

Participants reported that SARs and monitoring technologies could support older adults in managing their daily activities, which could help in reducing sedentary behaviors through reminders to be active or suggesting specific exercise routines and physical activities:

I wouldn’t want it to do anything physical...but I think mentally it could be good, you know, as a reminder thing. Like, doing some exercises or something like that, yeah.

Healthy individual

...well one of the things we could potentially look at doing would be to get a robot doing a kind of exercise routine with people.

Healthy individual

Alternatively, they may be a useful reminder of specific events or activities, medications, and maintaining social connections:

...a diary and, you know, you wake up in the morning and think “have I got anything today?” you know, you could ask...or press a button, “what’s on today?”

Low–socioeconomic status individual

...It could remind me, you know, “you need to call your aunty” or I think, if you could ask it, as I’ve said before, like a diary. You’d be able to go out, maybe to your garden, you know, and maybe it would do a conversation. Or “you need to take medicine.”

Individual with motor difficulties

##### Motivating and Stimulating Older Adults (SARs)

Participants reported that a possible consequence of using SARs was that they could make people lazier:

...the thing that worries me is that it could encourage you to become lazy It would be too easy to do nothing.

Low–socioeconomic status individual

However, it was suggested that the way in which SARs are implemented could have the opposite effect:

...the other issue, of course, is that they can encourage people to occasionally get up and do something rather than just sit.

Low–socioeconomic status individual

The activities suggested by SARs do not necessarily need to be physical but they can also involve other types of tasks:

I have a (older adult) friend whose main activity is jigsaw puzzles...I’m wondering whether something like this sat on the table next to him wouldn’t actually place the piece for him, but would point out the piece and where it might go.

Low–socioeconomic status individual

This may be especially true if motivation to complete daily activities is limited:

...sometimes, if I’m not very well I don’t always want to get out of bed...but maybe extra support...would help.

Individual with motor difficulties

...to me it would have to, sort of, push you type of thing.

Individual with motor difficulties

##### Providing Physical Assistance (SARs)

Older adults indicated that SARs were suitable aids designed to offer physical support in some scenarios—“...so when you can’t walk, it would walk for you, you know, but you’d be inside it or walking with it” (individual with motor difficulties)—or provide assistance in daily tasks requiring more strength than older adults may have, such as shopping, or tasks requiring grip strength—“I can drive in car and fetch shopping but its getting all the bags from the car” (individual with motor difficulties) and “I think the robot is beneficial because they can open things. Things like gripping and holding. That’s something that deteriorates with age” (individual with motor difficulties)—which could foster their independence:

...maybe it could help to be a bit more independent. I don’t need to have another person 24/7, but it could be a device that can help me with some tasks everyday.

Low–socioeconomic status individual

##### Continuous Monitoring of Health, Safety, and Activities (SARs and Monitoring Technologies)

Participants expressed that a strength of SARs and monitoring technologies is that they can support continuous care for older people:

...the side of looking after someone who does need 24/7 care, it’s a good thing. But there are a lot of things to take on board.

Low–socioeconomic status individual

This could assist carers in monitoring older adults’ whereabouts:

...she can have a tracking device just popped in her bag, and then with a smart phone someone can always know where she is. I think that might be a good idea. I mean, she lives on her own independently, but at least you can keep an eye on them.

Individual with motor difficulties

In addition, it could provide peace of mind for carers overnight:

I think there are benefits for carers in terms of if you are a carer, say at night time...Because it’s actually really difficult if you’re a carer. You never really sleep. You’re constantly on the alert in case the person you’re looking after is needing help.

Individual with motor difficulties

According to participants, the monitoring systems should encompass emergencies and risky situations alongside general monitoring for the benefit of both older adults and carers:

...you would know that she’s just safe. Or if something did happen, you’d know to go over to them.

Individual with motor difficulties

##### Social Support (SARs)

Participants emphasized that loneliness is a significant problem for older adults:

...if you’re looking at really old people that you’re trying to help, they probably wouldn’t have a lot of interaction. That’s what I find. They don’t have an awful lot of interaction.

Low–socioeconomic status individual

Consequently, SARs were identified as potential social partners to alleviate loneliness:

...you could say, well yeah I want to talk about [this topic] and it already knows about it so it can have a discussion, you see that? That would be very helpful.

Individual with motor difficulties

This could also be in addition to other daily tasks:

...it could be while you’re doing something as well, because it can follow you, so if you’re having a conversation, the cameras are pointed at you, so you can keep up with you and stuff.

Individual with motor difficulties

However, some participants expressed reservations about SARs replacing human interactions:

I don’t know if these would ever get to that point...It couldn’t ever be human, obviously.

Individual with motor difficulties

Moreover, an additional feature reported by participants related to loneliness was supporting older adults’ social connections and communication with others:

...if we had something like, say, Pepper, which has a screen on it as well. Or like the Turtle one back here that could almost be like a video conferencing robot.

Low–socioeconomic status individual

...we can have an exchange by texting...that’s quite a useful thing... [Healthy individual]

#### Perceived Barriers

##### Personal Factors (SARs and Monitoring Technologies)

The discussion highlighted various personal factors among participants that could pose challenges in using technologies and staying active. According to participants, personal motivation emerged as a crucial factor in maintaining activity levels:

I think it’s a bit dependent on yourself isn’t it, really. It’s how much you want to keep going. 

Individual with motor difficulties

Many participants identified physical difficulties as significant barriers that arise with aging and highlighted specific daily challenges:

It’s the ageing body, it gets harder. Physical activity gets harder, and you have to push yourself more. It’s quite easy when you’re 20 to go and run, but it’s harder when you’re old.

Individual with motor difficulties

I have trouble cleaning me windows and that; changing the bed because I get dizzy and lose my balance.

Individual with motor difficulties

In addition to physical challenges, participants discussed perceptual issues and cognitive difficulties:

Sight deteriorates. Even with glasses, it can still be a problem when you get older.

Healthy individual

I tend to just forget things until someone else tells me that I should do something.

Low–socioeconomic status individual

These factors may act as barriers, adding to the difficulties in adapting to and familiarizing themselves with new technologies:

Just as we’re older, um, we would find it more difficult to understand what we have to do.

Healthy individual

Participants also observed that these challenges may lead to stress due to cognitively demanding situations, a possible sense of lack of control, or fears of technology breaking down:

I can’t handle too many things at once sometimes. I’m afraid it overwhelms me.

Healthy individual

It’s got to be under my control.

Low–socioeconomic status individual

I’d be worried if it would break down. I’m always worried that my computer is not going to fire up or something. So I’d certainly been concerned about having one of these in the house.

Low–socioeconomic status individual

Participants acknowledged the need for support in using these technologies:

I would need help, obviously. Sons, daughters, anybody, but yes I would definitely.

Healthy individual

##### Economic Factors (SARs and Monitoring Technologies)

Participants expressed concerns about the cost of these technologies:

...one thing is, well to me, what’s the cost of these things going to be?

Individual with motor difficulties

...just how much is it going to cost, I can imagine it would be quite an expensive luxury.

Low–socioeconomic status individual

This suggests that the affordability of such technologies is a crucial factor to consider, raising questions about potential financial support or subsidies:

...but how would that be financed? Because I mean, a lot of old people, they hardly have money to pay their bills and put food on the table.

Low–socioeconomic status individual

Interestingly, participants suggested rental periods as a possible solution, expressing the following idea:

...the ideal thing would be able to ring a robot center and say, “oh right, I’m at so-and-so address, send me a robot” and then have it for a day.

Individual with motor difficulties

##### Privacy (SARs and Monitoring Technologies)

The participants’ statements underscored significant apprehensions surrounding the privacy implications of SARs and monitoring technologies. Concerns regarded the extent of personal information collected, questioning its storage, custodianship, and the entities involved:

...how much personal information, and how much is collected—where is it and who is dealing with it? That’s another story, and that’s a thought.

Individual with motor difficulties

For instance, participants pondered the potential future uses of these data:

How is it used in the future? How is your life insurance, health insurance, going to use this?

Individual with motor difficulties

The discussion highlighted the importance of consent in the context of tracking individuals, emphasizing that individuals must willingly agree to be tracked:

...they have to agree to it. You can’t track somebody if they don’t want to.

Healthy individual

##### Spatial Issues (SARs)

Older adults emphasized practical considerations and challenges associated with the deployment of these technologies within households, particularly in the context of navigating physical spaces:

...you’d have to have the room, like space, as well around your house for it to go in different rooms.

Low–socioeconomic status individual

Participants highlighted the need for obstacle detection and avoidance mechanisms to prevent collisions and property damage:

...well, it needs to sense, I suppose, where the barriers are...it would need to be able to get over them without tripping or getting stuck in a certain place or whatever. Yeah, so I think that is something you need to consider and think about. And of course, every house is different.

Individual with motor difficulties

...be able to sense objects around him so that, you know, it doesn’t run into tables and break things.

Healthy individual

#### Suggestions and Preferences

##### Number of Devices (SARs and Monitoring Technologies)

Participants discussed their preferences concerning the number of SARs and monitoring technologies at home. Divergent opinions emerged during the conversation, with some participants expressing a preference for all-encompassing, multitasking SARs or single monitoring sensors:

If they’re going to get one it’s got to do everything.

Low–socioeconomic status individual

However, a consensus on this viewpoint was not reached as other participants advocated for a more specialized approach, suggesting different technologies for distinct tasks:

I’m not sure it would be a single robot. You know, I think with everything we’ve got...you can’t have one that does everything, and that certain tasks are done by certain pieces of technology, you know.

Healthy individual

A nuanced perspective emerged, arguing that the ideal number of SARs and monitoring technologies should be contingent upon an individual’s specific circumstances, such as the following:

It’s like having a sensor in the living room in case you fall. But if you fall in the hallway, you haven’t got one, have you? So, depending on how badly you are or how incapable you are of doing things. It would depend on obviously the size of the house or the room. If you’re in a one-bedroom flat and it’s all on ground level you don’t need that much, do you? It depends on the situation of the particular person.

Healthy individual

##### Physical Characteristics (SARs)

Participants expressed a range of opinions regarding the physical characteristics of SARs, demonstrating diverse preferences and considerations. Some participants favored humanoid robots, appreciating the humanlike features:

I like that one as well [Pepper]. Its more connected to you as a human. You know, you feel like you could talk to it and it understand you more because it looks at you.

Healthy individual

In contrast, others leaned toward nonhumanoid designs, emphasizing their distinct robotlike appearance:

...it [TurtleBot] looks more like a robot than the others. It’s much more like an object.

Healthy individual

Practical considerations emerged as a unifying theme among participants, with agreement that the design of SARs should be task oriented:

...well it depends on what the task is its doing, doesn’t it? I mean, we assume a robot is in the form of a human being, but a lot of robots aren’t.

Individual with motor difficulties

...the consensus is that they don’t need to be humanoid. A box on the decks...could be quite configurable depending on who you are and what you want it to be. But there are limits...that’s why there might be a range of robots in a different environment.

Individual with motor difficulties

Participants also expressed a preference for smooth movements, emphasizing the importance of versatility in motion:

...it would have to move in every direction. Spin round, bend forward, reach up.

Individual with motor difficulties

An additional concern raised was discomfort with SARs staring, particularly when not interacting, highlighting the importance of social cues and behaviors in shaping participants’ comfort levels:

...well it just tends to stare at you a lot, and even when you’re not talking to him, his hands and arms and that are moving and it makes you think “oh, well what’s he going to do?”

Low–socioeconomic status individual

Regarding animal-like SARs, participants expressed the view that, if SARs are to have animal-like characteristics, they should be realistic, featuring fur and tactile qualities:

...it would need to have fur, erm, and be a bit more tactile.

Low–socioeconomic status individual

However, despite this acknowledgment of realism, participants tended to dislike the animal-like designs:

...well just because it’s a cute little thing that’s all—it’s more like a distraction.

Individual with motor difficulties

Participants also acknowledged that, while petlike robots could offer more than just the companionship of a dog or cat, they might be perceived more as toys:

...it gives them more than just a, you know, dog or cat, so I think there is some good but I think it would be more of a, um, well a toy perhaps.

Low–socioeconomic status individual

In fact, a noteworthy trend among older adults was the expressed preference for SAR designs that differed from toys:

It sort of looks like a toy [NAO], don’t it? I would imagine if that’s the same size a pepper, it would be more robotic, a bit more visual that it’s a robot more than a toy—only because of its size.

Individual with motor difficulties

This characteristic was deemed important as participants expressed the view that SARs should not resemble toys and should not be perceived as such by adults:

I think they can be nice, and cute, and not scary for children—they are like toys but they are not to us.

Individual with motor difficulties

##### Personalization and Remote Control (SARs)

The focus groups provided valuable insights into participants’ preferences and expectations for the customization of SARs. Participants emphasized the importance of adjustable volume, particularly for individuals with hearing difficulties:

...well something where you could turn it up, since I’m a bit deaf. There is those that are hard of hearing.

Low–socioeconomic status individual

The participants discussed the idea of customizable voice options, for instance, allowing users to choose between different genders and accents:

...yes, and then you can select whether you want a ladies voice or a gent, or a softer voice—American accent, Australian accent.

Individual with motor difficulties

This suggestion reflects the importance of the diverse preferences that users may have regarding the characteristics of SARs. In fact, the concept of customizable interactions based on individual preferences was also brought up:

I think this is something that can be customized, for some people who really love the interaction and then for those who don’t. If you like someone staring into your eyes it can do that but otherwise it can be a bit sideways maybe.

Healthy individual

Similarly, a participant stated the following regarding the information used by SARs to enhance user experience:

...it would give you information on what’s happening. But I suppose you would have to get used to it, wouldn’t you—customize him to your way of thinking.

Individual with motor difficulties

Participants highlighted the convenience of voice activation over buttons, aligning with the idea of making SARs user-friendly and accessible, allowing for multitasking and seamless interaction:

A voice activation would be easier. I’m not good with buttons.

Individual with motor difficulties

...it allows you to multitask, you know. You could be getting ready for work and you can chat at the same time, you know, “what’s the weather today?” you know, and you can still be getting ready.

Healthy individual

In addition, some participants raised concerns about batteries and expressed a preference for SARs with the capability to recharge themselves:

...if you’ve got someone with Alzheimer’s or limited ability to move, you’d need them to be self-charging.

Low–socioeconomic status individual

##### Use (Monitoring Technologies)

The discussion on monitoring technologies addressed wearable and environmental sensors. Participants highlighted the advantages of wearable sensors, noting that they can be used wherever the person is located:

I think it would be useful to wear something because then it doesn’t matter where you are, it will pick you up.

Healthy individual

However, concerns were raised about the potential for losing these sensors or forgetting to put them on:

...you still might lose it somewhere. Might end up wandering into our storage cupboard!

Low–socioeconomic status individual

I’d probably forget to put it; I don’t have a good memory so I’d probably forget to put it on.

Low–socioeconomic status individual

In contrast, these concerns did not apply to environmental sensors:

Something wearable is always with the person as long as they remember to put it on, but something in the room is always there, you know.

Healthy individual

Overall, participants reached a consensus that using both types of sensors would be more beneficial:

If you take it off because you’ve been in the bath and then forget to put it back on, it’s not helpful. If you have both, then if you fall or something, it would serve both purposes.

Healthy individual

Importantly, participants stressed the need for strategic placement of environmental sensors, prioritizing areas of higher risk for older adults:

...the bathroom is a really bad place to fall because there’s a lot of hard surfaces and if you slip on a wet patch or something, then you’re more likely to injure yourself.

Individual with motor difficulties

...on the stairs. I think it’s a good thing. Because if you, I think you’re on that surface, you’re more likely to fall.

Individual with motor difficulties

Participants also suggested that familiarity with sensor technology could contribute to its acceptance and integration into daily life:

I mean, if you’ve got a burglar alarm in your house, you’ve got sensors like that. I don’t look at mine. Once they’re there you should get used to it.

Low–socioeconomic status individual

#### Actual Use (SARs and Monitoring Technologies)

Some participants conveyed both their efforts and enthusiasm to keep up with technology:

I always try to keep up to date with technology. I’ve got two sons who ensure that they do keep up to date with it. Otherwise, there’s a gap between the two generations.

Low–socioeconomic status individual

...yes I would use it, definitely, because I like, you know, I like new technology.

Individual with motor difficulties

However, others expressed difficulty in doing so and a reluctance to embrace new technology:

I don’t think our generation is capable of doing it now. I think we’re too old.

Low–socioeconomic status individual

The belief in the future prevalence of these technologies was a common sentiment among participants, drawing parallels with other technologies:

I think these things are already, kind of, with us and probably are only going to increase as technology, you know, the rate at which technology has happened is, you know, only going to continue along that upward arc, I think.

Healthy individual

I think even if you think about phones. Not that long ago no-one had them and yet now people don’t go anywhere without them.

Individual with motor difficulties

They also speculated about the integration of SARs and monitoring technologies into health care:

...it is something that, you know, in the future, hospitals—doctors, physios, occupational, you know, people—will recommend and prescribe something like this.

Low–socioeconomic status individual

Importantly, participants emphasized their willingness to embrace the use of SARs and monitoring technologies if they proved helpful and could be incorporated into daily tasks:

...if I needed help I would use it.

Individual with motor difficulties

I’d want a robot to help me with all the things I need to do if I found I couldn’t do them myself. I would be reluctant, and probably a bit resistant but I’d want it to help me.

Healthy individual

I could see them being very helpful to lots of people—to be helpful it’s got to do the things you want it to do.

Healthy individual

### Statistical Analysis of Questionnaires

Spearman correlations indicated that older adults’ perceived well-being was associated with attitudes toward domestic robots, assessed using the MdRAS. Specifically, physical well-being was significantly associated with the sense of familiarity with (*r*=−0.409; *P*=.02) and interest in (*r*=−0.381; *P*=.03) SARs, as well as with perceived utility (*r*=−0.592; *P*<.001) and control (*r*=−0.559; *P*=.001). This suggests that, as a person perceives lower physical well-being, they report higher levels of familiarity, interest, perceived utility, and control related to SARs. Psychological well-being was significantly associated with a negative attitude toward SARs (*r*=−0.397; *P*=.02) and environmental fit (*r*=−0.394; *P*=.02), indicating that lower perceived psychological well-being is linked to a higher negative attitude and increased concerns for environmental fit. In addition, psychological well-being was positively associated with variety (*r*=0.553; *P*=.001), suggesting that people with lower psychological well-being prefer less variety in domestic SARs.

**Figure 2:**
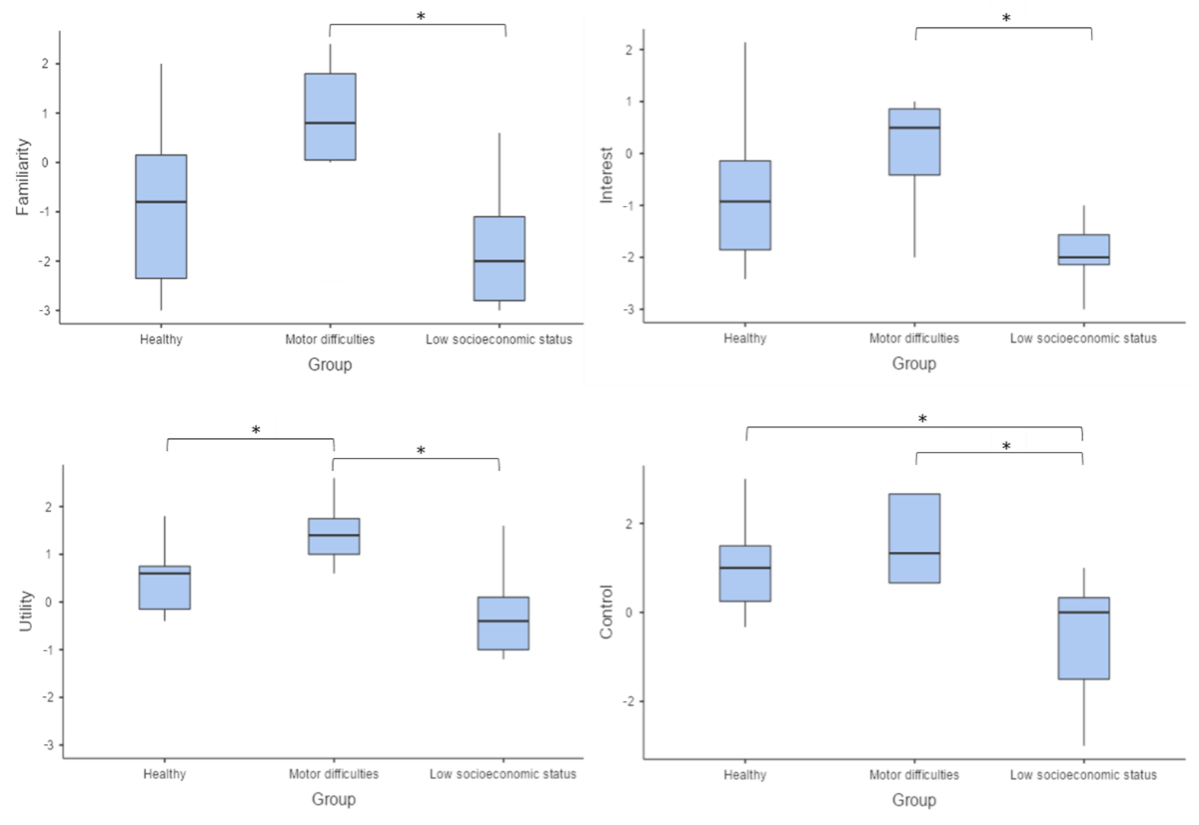
Box plot of the groups’ scores on the familiarity, interest, utility, and control subscales of the Multidimensional Robot Attitude Scale indicating the groups’ attitudes toward domestic social assistive robots. *P<.05.

The group comparison revealed significant differences in the dimensions assessed using the MdRAS: familiarity (χ^2^_2_=10.8; *P*=.005), where the group with motor difficulties showed higher perceived familiarity with SARs than the low–socioeconomic status group (*W*=−4.39; *P*=.005); interest (χ^2^_2_=12.0; *P*=.003), with the group with motor difficulties showing higher interest in SARs than the low–socioeconomic status group (*W*=−4.60; *P*=.003); utility (χ^2^_2_=14.5; *P*<.001), where the group with motor difficulties showed higher perceived utility of the SARs than the healthy (*W*=−3.88; *P*=.01) and low–socioeconomic status (*W*=−4.69; *P*=.003) groups; and control (χ^2^_2_=13.2; *P*=.001), where the low–socioeconomic status group perceived less control over SARs compared to the healthy group (*W*=−3.67; *P*=.02) and the group with motor difficulties (*W*=−4.91; *P*=.001). A graphical representation is shown in Figure 2.

Table 2 is a joint table of the qualitative and quantitative analyses. The results show that the group with motor difficulties showed significantly higher scores than the low–socioeconomic status group on the familiarity dimension assessed using the MdRAS, indicating a greater interest in using SARs for the related qualitative themes of motivation and social support. In contrast, participants from the low–socioeconomic status group reported lower perceived control over using SARs at home, highlighting that addressing the needs reported for personalization and remote control features may affect their acceptance. Although participants expressed a willingness to use these technologies if they were beneficial, the utility dimension of the MdRAS indicates that the group with motor difficulties perceived SARs as more useful, suggesting that they may have a higher acceptance of this technology.

## Discussion

### Principal Findings

The aim of this mixed methods study was to understand how healthy older adults, alongside those with motor difficulties and of a lower socioeconomic status, perceive the usefulness of SARs and monitoring technologies in the home to reduce sedentary behavior. This study identified key themes across 4 areas: the use of technology to support daily activities and reduce sedentary behavior, perceived barriers, preferences, and actual home technology use. In terms of using these technologies to assist with daily activities, participants highlighted their potential to help manage tasks, motivate and engage users, provide physical assistance, monitor health, and ensure safety. Perceived barriers included the need for social support, personal and economic limitations, privacy concerns, and spatial constraints. Suggestions focused on preferences for how many technologies they would like at home, desired features for SARs, the need for personalization and remote control, and how monitoring technologies should be used. Importantly, participants indicated that they would adopt these technologies if they effectively addressed their specific needs. These themes reflect how older adults suggest using these technologies to support their daily activities, as well as their preferences and concerns that should be addressed in the design and implementation of these solutions. Importantly, socioeconomic status and motor difficulties influenced perceptions. Older adults from lower socioeconomic backgrounds felt less control over SARs, highlighting the need to carefully consider these factors when designing for this group. In contrast, older adults with motor difficulties found SARs more useful, indicating that this group may have higher expectations and motivation to use such technologies. Notably, lower physical well-being was associated with greater interest in, familiarity with, and control over SARs, whereas lower psychological well-being was linked to more negative attitudes and concerns about environmental fit. Overall, this study underscores the importance of tailoring SARs to the specific needs of older adults to encourage adoption and improve their well-being.

Thematic analysis of the focus groups resulted in 14 themes outlining perceived needs and overall opinions on how these technologies may be used within older adults’ domestic environment. Participants indicated that SARs and monitoring technologies could help reduce sedentary behaviors by assisting in the management of daily activities and through providing motivation and stimulation. Many of the suggestions involved providing reminders, establishing routines or programs, and monitoring levels of sedentary behavior. The management of daily living activities poses a challenge for older adults and especially for health care professionals. Enhancing older adults’ ability to manage these activities has a significant impact on overall daily living [72].

Motivation is a key element when considering engagement in any physical activities, for instance, through highlighting potential benefits that the activity may entail [73]. When involved in the design of interventions or products to support physical activity, older adults tend to emphasize this motivational aspect [74]. Therefore, designing SARs and monitoring technologies aimed at motivating older adults to participate in physical activities could serve as a viable solution to mitigate sedentary behaviors at home. The combined results indicate that the group with motor difficulties scored significantly higher than the low–socioeconomic status group on the related quantitative measure of Familiarity, suggesting that this specific group has a greater interest in using SARs in relation to activity motivation.

In addition, older adults indicated that they viewed SARs as potential aids in providing physical assistance. Aging is characterized by a reduction in people’s physical strength and, thereby, a reduction in their capacity to perform ADLs [11,12]. Technologies could assist older adults with various tasks, catering to a wide range of specific needs such as helping with opening jars (perhaps to support cooking), transporting weights (eg, groceries), or physically assisting older adults (eg, help with walking). This was particularly important for the group with motor difficulties, who made most of the statements related to this theme.

A key perceived benefit offered by these technologies is their ability to monitor older adults’ health conditions, safety, and daily activities continuously, including overnight, which can be a particularly stressful time for caregivers. Risky situations such as falls are common in this population, especially in relation to gait during daily activities [75]. Thus, these are important strengths and considerations that monitoring technologies and SARs should address to support older adults at home. It is worth noting that the group with motor difficulties made a slightly higher proportion of statements related to this theme. We can assume that this aspect was of particular interest to them.

Loneliness is a reported issue among older adults [76], with previous work suggesting that socializing and communication are considered important daily activities for older adults [60]. This can be especially prevalent in groups of a low socioeconomic status [77], which was highlighted during our discussions with this group through their ideas of using SARs for social support. Finding solutions to cope with this condition may be challenging [78]; however, it has been suggested that SARs may reduce feelings of loneliness in older adults residing in care facilities [79] or enhance their social interactions [80]. Participants indicated a willingness to interact with SARs to alleviate loneliness or expressed interest in integrated functions that could enhance their social network. Participating in group activities and discussions has been shown to be beneficial for older adults’ social [81] and cognitive functioning [82]. Using SARs to facilitate this and as a tool for cognitive stimulation and discussion may be beneficial for older adults experiencing loneliness, which is one of the key future directions for research in this area. In this case, it is important to note that the group with motor difficulties presented significantly higher quantitative scores than the low–socioeconomic status group. This suggests that the group with motor difficulties find SARs more engaging in this role.

Several of the reported barriers to technology use for activity monitoring among the older population were related to personal factors such as personal motivation, with participants underscoring the significance of individual desire in maintaining activity levels regardless of technological intervention. Previously discussed physical challenges associated with aging, such as diminished strength and mobility, were also identified as notable obstacles alongside perceptual issues, such as deteriorating sight, and cognitive difficulties, such as forgetfulness. Moreover, cognitive challenges were expressed in concerns about the capacity to comprehend and effectively use these technologies, which may be perceived as overly complicated. Fear of technology breakdowns and a desire for control over the technology were expressed by many participants, indicating that the design and application of these technologies should be as user-friendly and simple as possible, thus avoiding additional cognitive demands and stress. Using these new technologies will subsequently require a learning process and dealing with issues such as software updates; this can pose cognitive and perceptual challenges to older adults [83], leading to increased stress as individuals adapt to these changes [84]. Participants acknowledged the need for support in using these technologies, suggesting that various forms of support should be considered as potential solutions. Future research should concentrate on addressing these challenges and determining the most effective solutions to facilitate older adults’ use of technologies.

When discussing the introduction of new technologies with end users, economic and privacy concerns are often raised [6,85]. Therefore, these should be carefully considered when making decisions about the selection and deployment of SARs and monitoring technologies. There was also a common emphasis on the importance of smooth movement within an environment, with participants also recognizing potential limitations due to physical space availability. This is of increased concern within a domestic environment, which could restrict the movement and subsequent usefulness of SARs. These considerations should be addressed before introducing SARs into the home environment to ensure a suitable fit.

Discussions regarding the number of sensors and SARs to use in one setting were pragmatic, indicating that the number of sensors and SARs should depend on the individual circumstances. This was also true when discussing the preferred characteristics of SARs; however, some similarities emerged, such as the reduced acceptance of animal-like robots and suggestions to avoid SARs that could be perceived as toys. Moreover, a preference for personalizing certain characteristics and being able to control SARs remotely was expressed. In this regard, the quantitative analysis indicated that participants from the low–socioeconomic status group tended to feel that they had less control over the use of SARs at home. This suggests that addressing this need could be a crucial consideration during the design process for this group. Interestingly, participants accepted both environmental and wearable technologies. Despite reporting the common strengths and weaknesses associated with each type of sensor [60], they indicated that using both types would be beneficial. It is important to use sensors in situations or areas in which there is a higher risk for older adults, such as those where falls are more probable. Finally, participants indicated that they would use SARs and monitoring technologies if they were perceived as helpful, confirming previous results [60]. Notably, quantitative results indicated that the group with motor difficulties had a higher perception of the utility of SARs at home, suggesting that they tend to view this technology as more helpful compared to the other groups.

These results align with other evidence suggesting SARs as potential tools to provide coaching, monitoring, and companionship [86] and promote changes in daily routines [87]. In addition to other important key points emerging from the analysis, considering 3 different groups characterized by different socioeconomic levels and physical difficulties further developed our understanding of the perspectives on these technologies within a wider category of older adults. Perceived physical and psychological well-being are associated with various dimensions regarding SARs. When an older adult perceives themselves as more physically impaired, they are more likely to feel a sense of familiarity with and interest in SARs. This perception is coupled with the perceived utility and the ability to control SARs. On the other hand, if an older adult perceives a lower psychological wellness, they may have more reservations about SARs due to a general negative attitude and concerns regarding the environmental fit of the SARs. Interestingly, this was associated with less interest in SAR variety. Several factors, as indicated in the literature, are associated with the intention to use SARs or their acceptability among older adults [88,89]. However, to the best of our knowledge, this is the first study indicating that specific well-being domains—psychological and physical—play a pivotal role in attitudes toward SAR use at home. This is noteworthy considering that most SAR use is related to health [88]. Group comparisons also yielded interesting insights, largely aligning with the associations discussed. The group with motor difficulties perceived SARs as more familiar and expressed greater interest than the low-income group. In addition, the group with motor difficulties considered SARs to be more useful compared to the other groups. On the other hand, participants of a low socioeconomic status indicated less perceived control over SARs compared to the other groups. This aligns with a recent survey indicating that people of a low socioeconomic status are less supportive of technologies and SARs [90]. In this regard, our results suggest a connection with the perceived barriers associated with these technologies as the participants from the low–socioeconomic status group tended to report the highest percentages of statements related to all the perceived barriers identified in the analysis, including personal factors, economic considerations, privacy concerns, and space issues. This suggests that, for this specific category, it may be crucial to demonstrate the usefulness of SARs and monitoring technologies and address older adults’ needs. Importantly, older adults with physical difficulties had more positive attitudes toward SARs at home compared to the other groups. These insights highlight their tendency to be more accepting of SARs in their homes for support, aligning with the findings obtained from the thematic analysis.

### Limitations

Despite yielding interesting results, this study has certain limitations. The limited sample sizes of the 3 groups for quantitative analysis represent a limitation of the study; thus, the statistical results should be interpreted with caution. Therefore, the results of the quantitative statistical analysis need to be replicated, and future studies should confirm these findings with larger sample sizes. Demographic variations and characteristics specific to older adults may influence their needs and opinions. For instance, our results suggest that psychological well-being significantly influences attitudes toward SARs. Therefore, future studies should encompass older adults with psychological difficulties, such as depression, for a more comprehensive understanding. Cognitive and sensory challenges among participants were not evaluated, which should be considered for future research. This study did not consider other demographic characteristics of older adults, such as gender and ethnicity, which should be included in future studies.

### Conclusions

The participants indicated that SARs and monitoring technologies could help reduce sedentary behaviors and assist in the management of daily activities. This study highlights the importance of considering the functional and socioeconomic characteristics of older adults as future users of SARs and monitoring technologies to promote widespread adoption within this population and improve well-being. Older adults with different characteristics and backgrounds may have varying attitudes and needs, which the design and implementation of technologies should take into account.

## Appendix

Multimedia Appendix 1 Checklist for Reporting Results of Internet E-Surveys.

## Abbreviations

ADL: activity of daily living
MdRAS: Multidimensional Robot Attitude Scale
SAR: social assistive robot

## Data Availability

The datasets generated during and analyzed during this study are available in the Zenodo repository [71].
