# Supplementary material for: Social Robots and Sensors for Enhanced Aging at Home: Mixed Methods Study With a Focus on Mobility and Socioeconomic Factors
Source: JMIR Aging. 2024 Nov 25;7:e63092. doi: 10.2196/63092 (PMC11629043; doi:10.2196/63092)
Supplement: Multimedia Appendix 1 [file aging_v7i1e63092_app1.docx]

| Item Category | Checklist Item | Explanation |
| --- | --- | --- |
| Design | Describe survey design | A convenience sample of older adults (aged ≥ 65 years) were recruited and divided into one of three categories: 1) low socio-economic; 2) motor difficulties; 3) healthy |
|  | IRB approval | Study has been approved by the institutional human research ethics committee (ID: 1726544) |
|  | Informed Consent | Participants were provided details of the survey within the recruitment phase, as well as at the beginning of the survey. This included the expected completion time (30-40 minutes), an outline of the studies purpose, details relating to data storage. Contact details for the research team were also provided. |
|  | Data Protection | Only participants who expressed an interest in the study were provided a link to the survey, and data was stored in a password protected file accessible only to the research team. |
| Development and pre-testing | Development  and testing | The survey was created using JISC software. Volunteers tested the survey prior to it being released to check for usability and addressing any technical issues. |
| Recruitment process and description of the sample having access to the questionnaire | Open survey versus closed survey | This was an open survey in that it was not password protected but was only made available to those wishing to participate. |
|  | Contact mode | Potential participants were recruited through social media, email lists and advertisement from charity groups, care homes and universities. |
|  | Advertising the survey | The survey was advertised via university mailing lists, social media and physical posters as part of the overall study recruitment. |
| Survey administration | Web/E-mail | Participants were provided a link to the web-based survey via email. The software used (JISC) allows responses to automatically downloaded. |
|  | Context | The survey was only sent to individuals participating in the overall study (including focus groups). It was not placed on an open website to be filled in and therefore had a reduced risk of bias. |
|  | Mandatory/voluntary | Voluntary |
|  | Incentives | The participants were reimbursed for their travel expenses |
|  | Time/Date | June to September 2023 |
|  | Randomization of items or questionnaires | No randomisation or alteration were made as this was a web-based version of validated questionnaires. |
|  | Adaptive questioning | No adaptive questioning as this was a web-based version of validated questionnaires. |
|  | Number of Items | 70 items were included; 12-Item Short Form Health Survey (12 items), Muti-dimensional Robot Attitude Scale (49 items). Demographic questions. |
|  | Number of screens (pages) | The questionnaire was distributed across five screens (pages). |
|  | Completeness check | Completeness checks were done following submission. All questions were mandatory and could not be skipped. |
|  | Review step | Participants were able to adjust their answers via a “back” if they had moved onto another section of the survey, or by simply selecting a different option before continuing to another section. |
| Response rates | Unique site visitor | *N/A* |
|  | View rate (Ratio of unique survey visitors/unique site visitors) | *N/A* |
|  | Participation rate (Ratio of unique visitors who agreed to participate/unique first survey page visitors) | *N/A* |
|  | Completion rate (Ratio of users who finished the survey/users who agreed to participate) | 100% |
| Preventing multiple entries from the same individual | Cookies used | *N/A* |
|  | IP check | *N/A* |
|  | Log file analysis | Participants were required to provide a unique identifier based on a set of questions when completing the survey which were checked prior to analysis. In cases where there were multiple entries for one identifier, the most recent submission was used in analysis |
|  | Registration | *N/A* |
| Analysis | Handling of incomplete questionnaires | Only fully completed questionnaires were included in the analysis. |
|  | Questionnaires submitted with an atypical timestamp | *N/A* |
|  | Statistical correction | No correction has been performed. |
